# Visual illusion in male self-assessment of penile dimensions: a clinical study on penile length perception bias between flaccid and erect states

**DOI:** 10.1093/sexmed/qfaf068

**Published:** 2025-09-10

**Authors:** Zhongjie Zheng, Yan Chen, Wei Zhang, Qianxi Chen, Zhen Liu, Eric Chung, Kai Hong, Haocheng Lin

**Affiliations:** Department of Urology, Peking University Third Hospital, Peking University, Beijing 100191, China; Center for Reproductive Medicine, Department of Obstetrics and Gynecology, Peking University Third Hospital, Peking University, Beijing 100191, China; Department of Urology, Peking University Third Hospital, Peking University, Beijing 100191, China; Center for Reproductive Medicine, Department of Obstetrics and Gynecology, Peking University Third Hospital, Peking University, Beijing 100191, China; Department of Urology, the People's Liberation Army No. 92493 Hospital (PLA No. 92493 Hospital), Huludao 125000, China; Department of Urology, Peking University Third Hospital, Peking University, Beijing 100191, China; Center for Reproductive Medicine, Department of Obstetrics and Gynecology, Peking University Third Hospital, Peking University, Beijing 100191, China; Department of Urology, Peking University Third Hospital, Peking University, Beijing 100191, China; Peking University Health Science Center, Peking University, Beijing 100191, China; Department of Urology, Princess Alexandra Hospital, University of Queensland, Brisbane, QLD 4000, Australia; AndroUrology Centre, St. Andrew’s War Memorial Hospital, Brisbane, QLD 4000, Australia; Department of Urology, Peking University Third Hospital, Peking University, Beijing 100191, China; Center for Reproductive Medicine, Department of Obstetrics and Gynecology, Peking University Third Hospital, Peking University, Beijing 100191, China; Department of Urology, Peking University Third Hospital, Peking University, Beijing 100191, China; Center for Reproductive Medicine, Department of Obstetrics and Gynecology, Peking University Third Hospital, Peking University, Beijing 100191, China

**Keywords:** penis, length, penile dimensions, self-perception bias, penile lengthening ratio

## Abstract

**Background:**

Despite the significance of penile dimensions in male health and self-perception, there is a lack of population-specific references and understanding of self-assessment biases.

**Aim:**

To establish population-specific references and investigate self-assessment biases in penile dimensions.

**Methods:**

A single-center cross-sectional study (2024-2025) prospectively enrolled 342 Chinese males. Standardized measurements of flaccid and stretched lengths were performed by a trained andrologist. Participants were asked to report their perceived erect penile length, and stratified into three mutually exclusive groups: (1) accurate estimation (AE), where self-reported lengths = stretched lengths; (2) overestimation (OE), where self-reports > stretched lengths; and (3) underestimation (UE), where self-reports < stretched lengths. The penile lengthening ratio (PLR) was calculated as (stretched − flaccid length)/flaccid length. Statistical analyses included paired *t* tests and one-way analysis of variance for multigroup comparisons.

**Outcomes:**

Mean flaccid and stretched lengths, self-reported lengths, and the distribution of estimation groups were determined.

**Results:**

Mean flaccid and stretched penile lengths were 7.27 ± 1.60 and 11.89 ± 1.57 cm, respectively. Self-reported erectile lengths (12.81 ± 1.85 cm) significantly exceeded measured values (*Δ* = 0.92 cm, *P* < .001), with 72.81% of the participants overestimating their erectile length. OE participants exhibited greater flaccid (7.46 ± 1.64 vs 6.74 ± 1.39 cm, *P* < .05) and stretched lengths (12.01 ± 1.47 vs 11.46 ± 1.69 cm, *P* < .05) than AE participants. UE individuals showed paradoxically higher stretched lengths (13.50 ± 2.38 vs 11.46 ± 1.69 cm, *P* < .05) and PLR (97 ± 36% vs 71 ± 14%, *P* < .05).

**Clinical Implications:**

These findings provide critical references for clinical counseling on penile size and perioperative doctor-patient communication, potentially alleviating patient anxiety stemming from cognitive biases to a certain extent.

**Strengths and Limitations:**

The strengths include standardized measurements and a clear classification of estimation group. The limitations include incomplete baseline data (lacking penile circumference, smoking history, etc.), single-center small-sample bias, inevitable selection bias, and absence of partner satisfaction data and validated assessments.

**Conclusion:**

This study revealed that self-reported erect lengths among adult males were significantly longer than clinician-measured stretched lengths. OE participants accounted for more than 70% of the sample, while UE participants tended to have a greater PLR. The research provides reference ranges for flaccid and stretched penile lengths in Chinese males, offering objective data to support clinical counseling and surgical communication. This not only alleviates patient anxiety rooted in cognitive biases but also elucidates the potential association between penile size misperceptions and PLR.

## Introduction

As a defining morphological feature of male secondary sexual characteristics, penile dimensions can have substantial sociocultural implications and clinical significance to men. Accumulating evidence indicates that penile size not only serves as a sociocultural symbol of virility and masculinity, but also exerts profound impacts on male psychological well-being.^1^^,^^2^ A striking “penile size perception paradox” has been observed cross-culturally where literature reports that while 85% of females express satisfaction with their partner’s genital dimensions, 45-68.3% of males demonstrate clinically significant penile size anxiety.^3–6^ This marked discrepancy between objective assessments and subjective perceptions underscores the variations between what females and males perceive as relevant and the profound influence of sociocultural constructs on male body image formation.

From a clinical perspective, penile size–related distress has emerged as a critical issue in contemporary men’s health management. A nationwide survey encompassing 25 000 heterosexual American males revealed that 45% of the participants sought penile enlargement, with 30% of these individuals meeting the diagnostic criteria for body dysmorphic disorder.^7^ Such pathological anxiety frequently originates from distorted perceptions of idealized masculine archetypes, consequently driving an increased demand for unnecessary penile augmentation procedures.^4^^,^^8^ These findings highlight the urgent need for establishing evidence-based normative references for penile dimensions through standardized measurement protocols.^9^^,^^10^

Current research limitations primarily manifest in two domains: first, a concern regarding actual sample representation, as most studies employ self-reported measurements from volunteer cohorts, introducing substantial selection bias,^5^ and second, inconsistent conclusions often exist regarding correlations between penile dimensions and anthropometric parameters such as stature and digit length. Notably, while early studies reported median self-measured erect penile lengths of 15-16 cm, subsequent studies utilizing standardized stretched penile measurement techniques suggest potential overestimation in these initial findings.^4^^,^^5^^,^^10^ The observed methodological disparities emphasize the necessity for measurement standardization. Numerous factors such as temperature, sexual arousal, and sympathetic tone can significantly affect the actual penile size at the time of physical examination. Furthermore, there are inter- and intraobserver variations and underestimation of the actual penile measurements. Current consensus recognizes stretched (flaccid) length measurement as the gold standard, due to high-evidence-level meta-analysis demonstrating nearly identical results with erect length.^4^^,^^10^

In the context of penile size research, the penile lengthening ratio (PLR) has emerged as a significant parameter. The PLR is calculated as the percentage increase in penile length from the flaccid state to the stretched or erect state. Understanding PLR not only helps in quantifying the physiological variability in penile length changes among individuals but also has potential implications for clinical practice. In cases of suspected penile dysmorphia or when evaluating the effectiveness of penile lengthening procedures, the PLR can serve as a valuable metric. For example, in patients considering penile lengthening surgery, preoperative assessment of their natural PLR might provide insights into the potential achievable results of the surgery. Additionally, in research on body image disorders related to penile size, the PLR can be used to better understand how the perceived discrepancy between flaccid and erect lengths impacts an individual’s psychological well-being.

Is there a widespread phenomenon of overestimation in self-assessed penile length among Chinese adult males? And is there a correlation between this overestimation bias and actual penile size, as well as the PLR? This single-center cross-sectional study aims to establish standardized reference ranges for flaccid and stretched penile lengths (SPLs) in Chinese males, providing objective data to support clinical diagnosis of “penile dysmorphic disorder” and alleviate patients’ anxiety from cognitive biases. It also seeks to reveal the patterns of deviation between self-reported and measured penile lengths, explaining why patients with penile prosthesis often complain of “perceived shortening after surgery”— preoperative self-overestimation may lead to postoperative perceptual discrepancy, offering a theoretical basis for presurgical doctor-patient communication and postsurgical psychological intervention.

## Methods

This prospective observational study was conducted at Peking University Third Hospital between December 2024 and March 2025, involving 342 Chinese males who had previously attended the andrology clinic of the Reproductive Medicine Center. Participants were recruited during routine andrology consultations. The exclusion criteria included the following: patients < 18 years, chronic erectile dysfunction (a disease course of >6 months and International Index of Erectile Function-5 score < 21), prior pelvic surgery, secondary hypogonadism, and penile pathologies or congenital abnormalities, with additional exclusion criteria as previously documented.^3^^,^^11^ Sample size was initially set at 350 to ensure sufficient statistical power, referencing similar studies on penile morphology in Asian populations.^12^^,^^13^

The baseline data collected included age, height, weight, ethnic background, and self-reported erect penile length. All the participants received standardized instructions on penile measurement protocols for both flaccid and maximally stretched states. Measurements were performed by a single trained andrologist in a controlled environment maintained at 25 °C to ensure consistency. Subjects assumed an upright stance while a disposable millimeter-graduated paper ruler was applied along the dorsal penile surface. Penile length was measured from the base of the pubic symphysis bone at the start of the penile skin insertion to the glans tip under both flaccid and stretched conditions.

Anthropometric parameters (age, height, weight) were recorded for all the participants, with body mass index (BMI) calculated as kilogram per meter squared. Penile dimensions were quantified as flaccid penile length (FPL) and SPL. The PLR was derived using the formula: PLR = (SPL − FPL)/FPL.^14^^,^^15^

Participants completed penile length assessment where they reported perceived penile lengths, which were later compared to objectively measured stretched lengths using a standardized tape protocol, and the measured lengths were primarily rounded to the nearest centimeter. Based on this comparison, participants were stratified into three mutually exclusive groups: (1) accurate estimation (AE), where self-reported lengths = stretched lengths; (2) overestimation (OE), where self-reported lengths > stretched lengths; and (3) underestimation (UE), where self-reported lengths < stretched lengths.

### Statistical analysis

Continuous variables are presented as mean and standard deviation (SD) and were compared by Student’s independent *t* test or the Mann-Whitney *U* test based on their normal or not-normal distribution, respectively (the normality of variables’ distribution was tested by the Kolmogorov-Smirnov test). Categorical variables were tested with the chi-square test. Linear regression was used to evaluate whether the height can be associated with the penile length and circumference. All the statistical analyses were completed using GraphPad Prism version 9 (GraphPad Software, California, United States, www.graphpad.com). For all statistical comparisons, a significance level of *P* < .05 was considered to show differences between the groups by Wilcoxon’s signed rank test.

## Results

### Study selection and post hoc power analysis

**Figure 1:**
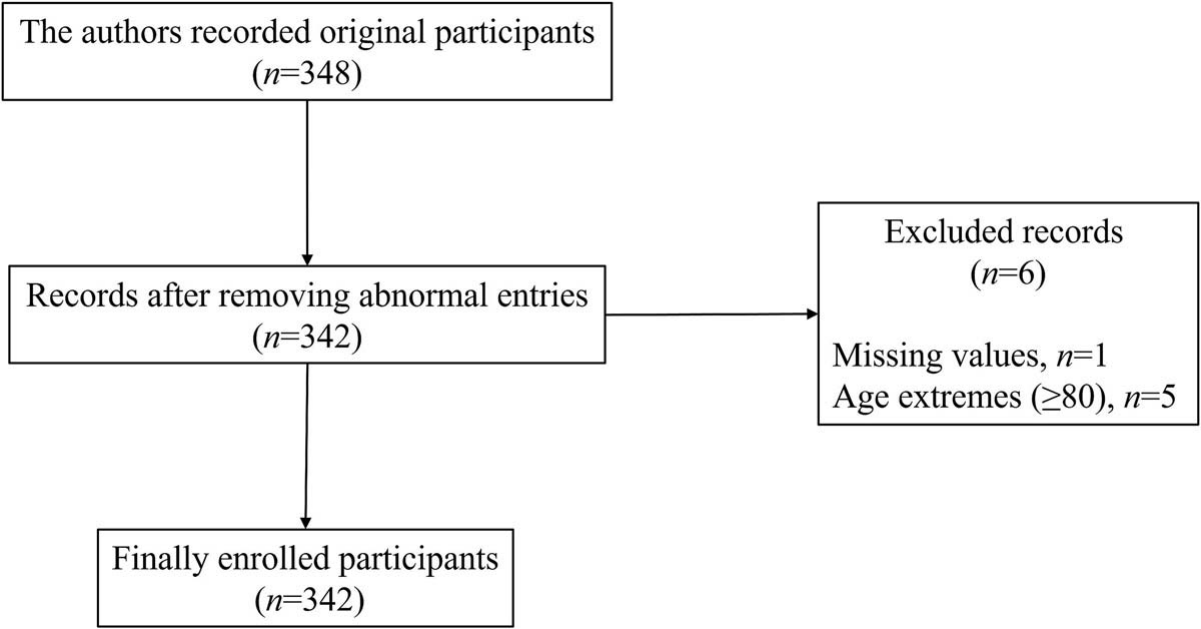
Flowchart of the studies included in the final research.

Figure 1 provides a flowchart of the study and the number of participants who were screened for eligibility and subsequently excluded from or included in the final research.

A retrospective power analysis was conducted using G^*^Power 3.1.^16^ A post hoc power analysis confirmed that 342 participants provided 82% power to detect a moderate effect size (*d* = 0.5) at *α* = 0.05, supporting the validity of our findings.

### Synthesis of results

The baseline characteristics of the population are shown in Table 1. The mean (standard deviation [SD]) age was 31.85 (5.88) years, mean (SD) height was 175.8 (5.64) cm, mean (SD) weight was 80.49 (13.96) kg, and mean (SD) BMI was 26.03 (4.16) kg/m^2^. Mean (SD) flaccid penis length was 7.27 (1.60) cm, mean (SD) stretched penis length was 11.89 (1.57) cm, and mean (SD) self-reported penis length was 12.81 (1.85) cm.

**Table 1:** Epidemiological data of the cohort.

**Volunteers, N = 342**	
Age (years), mean (SD)	31.85 (5.88)
Height (cm), mean (SD)	175.80 (5.64)
Weight (kg), mean (SD)	80.49 (13.96)
BMI (kg/m^2^), mean (SD)	26.03 (4.16)
Flaccid length (cm), mean (SD)	7.27 (1.60)
Stretched length (cm), mean (SD)	11.89 (1.57)
Self-reported length (cm), mean (SD)	12.81 (1.85)
Penile lengthening ratio (%), mean (SD)	72 (16)
**Penile length perception bias, n (%)**	
Accurate estimation	89 (26.02)
Overestimation	249 (72.81)
Underestimation	4 (1.17)

Abbreviations: SD, standard deviation; BMI, body mass index.

**Figure 2:**
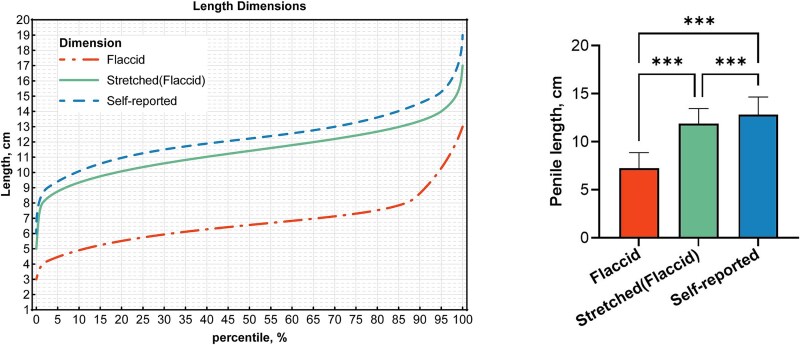
Penile length nomogram with comparative analysis of mean values. Data are expressed as the mean ± SD. Statistical analysis was performed using an unpaired *t* test. ^*^P < .05, ^**^P < .01, and ^***^P < .001; ns: not significant.

The nomogram and paired samples *t*-test results demonstrated that the mean stretched length was significantly greater than the flaccid length, and the mean self-reported length was notably higher than the measured stretched length (*P* < .001). This indicates that these male patients tended to overestimate their penile length by approximately 1 cm on average (Figure 2).

**Figure 3:**
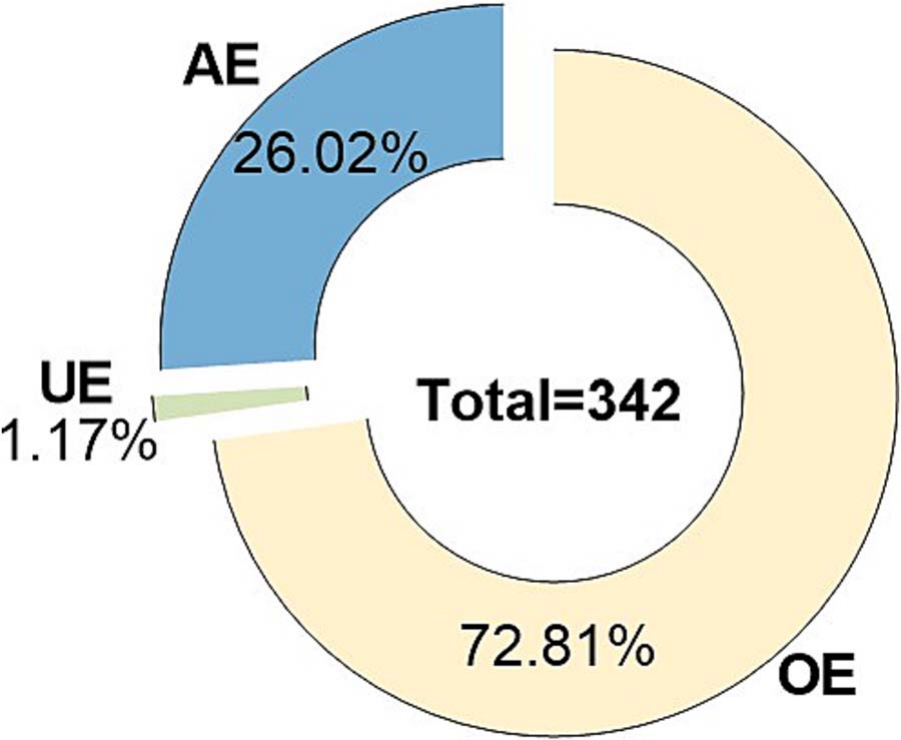
Pie chart of the proportional distribution across stratified groups. AE: accurate estimation; OE: overestimation; UE: underestimation.

The percentage distribution stratified by self-reported accuracy is presented in Figure 3.

**Figure 4:**
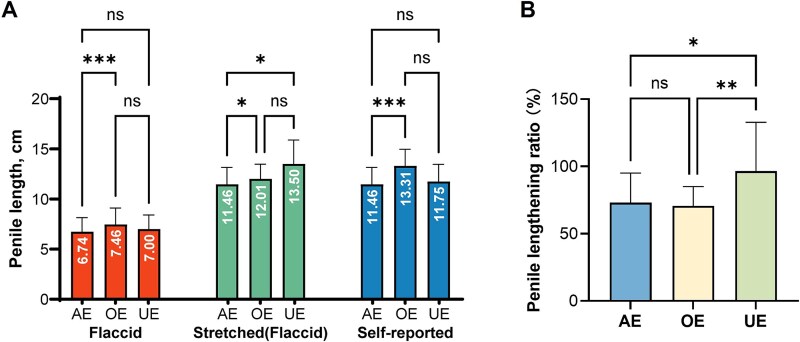
Comparison of penile length and penile lengthening ratio (PLR) in different groups. (A) Comparison of flaccid length, stretched length, and self-reported length across groups. (B) Comparison of PLR between groups. Data are expressed as mean ± SD. Statistical analysis was performed using an unpaired *t* test. ^*^P < .05, ^**^P < .01, and ^***^P < .001. NS: not significant; AE: accurate estimation; OE: overestimation; UE: underestimation.

Table 2 presents the baseline characteristics of populations stratified into AE, OE, and UE groups. Our analyses revealed that males in the OE group exhibited significantly greater mean values in both flaccid and SPLs compared to the AE group (*P* < .05), though no statistically significant differences were observed in median measurements. Notably, the UE group demonstrated no significant deviations in either mean or median flaccid length relative to both the AE and OE groups. However, their SPL showed statistically elevated mean and median values versus the AE group (*P* < .05) (Figure 4A). Further comparative analysis of the PLR demonstrated that the UE group had significantly higher PLR values than both the OE and AE groups (*P* < .05), while no significant differences were detected between the OE and AE groups in PLR measurements (Figure 4B).

**Table 2:** Baseline characteristics of the accurate estimation (AE), overestimation (OE), and underestimation (UE) groups in the study.

**Volunteers, N = 342**	**AE, N = 89**	**OE, N = 249**	**UE, N = 4**	***P* value**
Age (years), mean (SD)	31.96 (6.75)	31.86 (5,59)	29.00 (2.45)	.618
Height (cm), mean (SD)	175.60 (5.33)	175.80 (5.79)	173.80 (1.71)	.738
Weight (kg), mean (SD)	82.22 (14.21)	79.82 (13.91)	83.50 (8.96)	.344
BMI (kg/m^2^), mean (SD)	26.58 (3.88)	25.80 (4.27)	27.65 (2.85)	.235
**Penile dimensions (cm), mean (SD)**				
Flaccid length^**^	6.74 (1.39)	7.46 (1.64)	7.00 (1.41)	.001
Stretched length^**^	11.46 (1.69)	12.01 (1.47)	13.50 (2.38)	.002
Self-reported length^***^	11.46 (1.69)	13.31 (1.65)	11.75 (1.71)	<.001
Penile lengthening ratio (%)^**^	73 (22)	71 (14)	97 (36)	.008
**Penile Dimensions (cm), median (IQR)**				
Flaccid length	7 (6-7)	7 (6-8)	7.5 (5.5-8)	NA
Stretched length	12 (10-12)	12 (11-13)	12.5 (12-16)	NA
Self-reported length	12 (10-12)	13 (12-14)	11.5 (10.25-13.5)	NA

Abbreviations: SD, Standard deviation; BMI, Body mass index; IQR, interquartile range; NA, not applicable.

## Discussion

The current literature demonstrates a conspicuous absence of systematic investigations addressing both penile morphometric measurements and self-assessment accuracy among Chinese males. Our pioneering study provides normative references for Chinese adult males, with mean flaccid length at 7.27 cm (SD ±1.60), stretched length at 11.89 cm (SD ±1.57), and a PLR of 72% (95% CI 56-88). Interestingly, comparative analysis revealed a systematic overestimation bias in self-reported dimensions, with 72.81% of the participants exceeding their measured stretched length by a mean of 0.92 cm. The tripartite classification model (AE/OE/UE groups) yielded further interesting observations that OE subjects demonstrated significantly greater flaccid dimensions versus their AE counterparts (*Δ* = 0.72 cm, *P* < .001), suggesting this physical characteristic may contribute to their perceptual overestimation. Conversely, UE subjects showed no flaccid length advantage but paradoxically exhibited greater stretched lengths than their AE peers (*Δ* = 2.04 cm, *P* < .05), suggesting dissociation between anatomical reality and self-perception. Notably, the PLR was inversely associated with self-assessment accuracy, with individuals exhibiting higher PLR values showing increased likelihood of underestimation.

The terms “grower” and “shower” historically describe penile dimensional changes between flaccid and erect states, defined as ≥4 cm versus <4 cm erectile length increment respectively.^17^ A 2018 clinical study (N = 274) on erectile dysfunction patients revealed a 26% prevalence of growers compared to 74% showers.^17^ While no clinically significant differences in sexual function were observed between phenotypes, our preliminary findings suggest potential psychosexual correlations that need further validation.

Recent years have witnessed systematic investigations into penile dimensions across global populations, primarily focusing on length and circumference analyses.^1^^,^^3^^,^^12^^,^^13^^,^^18^ Our analysis revealed consistency with selected studies reporting SPL ranges of 10-13 cm among East/South Asian populations.^1^ However, substantial discrepancies emerge when comparing these findings to numerous reports documenting higher mean stretched lengths (15-16 cm) in other demographic groups, highlighting ongoing debates regarding anthropometric applicability across different ethnicities.^1^^,^^3^^,^^5^^,^^10^ Crucially, these morphometric references prove vital for clinical interventions addressing micropenis misconceptions, penile size anxiety, and body dysmorphic disorder—conditions where targeted sexual health education and psychological counseling demonstrate therapeutic efficacy.^4^^,^^5^ Our evidence-based measurements (flaccid: 7.27 cm; stretched: 11.89 cm) provide valuable references to inform clinical counseling strategies and guide psychosexual education initiatives, especially in the Chinese population. This finding corroborates a systematic analysis by Wang^15^ reporting Chinese penile dimensions of 7.42 ± 0.95 (flaccid) and 12.42 ± 1.63 cm (erect).

The existing literature suggesting male tendency toward penile size underestimation appears contradictory to our findings.^3^^,^^5^ This discrepancy arises from differing operational definitions: previous studies primarily addressed dissatisfaction-driven underestimation (perceived inadequacy), whereas our research specifically evaluates perceptual accuracy (measured vs self-reported dimensions). Crucially, body image perception and sexual satisfaction demonstrate significant clinical correlations, with size-related anxiety potentially exacerbating psychogenic erectile dysfunction.^5^^,^^8^^,^^19^^,^^20^ Patients harboring micropenis misconceptions frequently exhibit decreased sexual frequency and impaired ejaculatory control, underscoring the diagnostic necessity of evidence-based size references.^4^^,^^20^ Notably, contemporary surveys reveal hierarchical dissatisfaction patterns: flaccid appearance (27%), erect length (19%), and erect girth (15%).^21^ Clinically, we express heightened concern regarding the risks associated with size overestimation and postoperative perception discordance. Patients undergoing penile procedures might inappropriately attribute surgical outcomes to length reduction, emphasizing the imperative for preoperative standardized measurements (flaccid/stretched/erect) and postoperative reassessment protocols to mitigate medicolegal disputes.^22^

The belief that penile size serves as a primary marker of masculinity is profoundly influenced by the absence of formal sexual education in many regions. Globally, adolescents often rely on pornography as a surrogate source of sexual knowledge due to inadequate school-based or familial education.^23–25^ Studies have shown that exposure to pornographic media, which frequently exaggerates penile dimensions as a symbol of virility, reinforces misconceptions about sexual function and masculinity.^26^ For instance, longitudinal research indicates that young males who consume explicit content are more likely to associate penile size with self-worth, a correlation that persists in the absence of evidence-based sexual health education. This underscores the need for comprehensive educational initiatives to counteract these distortions and foster healthier body image perceptions.

This study has several limitations that should be acknowledged. First, the baseline data collection was incomplete. Penile circumference was not measured, and the medical histories related to other systems were not queried. Whether the patients had undergone circumcision was not recorded. Additionally, we did not inquire whether the patients had ever self-measured their penile length and what the measurement results were. These unrecorded baseline data may be crucial for a more comprehensive understanding of the study subjects. Secondly, the study was inevitably affected by selection bias. The participants were recruited from outpatient clinics, resulting in a limited sample size and data sourced from a single center. This restricts the representativeness of the data. Although patients up to 80 years old were included, the patient population predominantly consisted of young and middle-aged men seeking pre-pregnancy examinations or consultations regarding fertility and sexual function issues. Consequently, the vast majority of participants were of childbearing age, which further contributed to the selection bias. Moreover, our measurement approach involved rounding the data to the nearest centimeter, and we did not consider a tolerance range during classification, potentially influencing the classification outcomes. Thirdly, we did not investigate the satisfaction of the participants’ partners, nor did we conduct sufficient stratified analyses among different patient types. These omissions limit the in-depth exploration and utilization of the data. Finally, although previous research suggests that SPL is comparable to fully erect length, it cannot be considered a perfect substitute for actual erect length.^9^

## Data Availability

The data that support the findings of this study are available from the corresponding authors upon reasonable request.
